# Prognostic role of ABO blood type in patients with extranodal natural killer/T cell lymphoma, nasal type: a triple-center study

**DOI:** 10.1186/s40880-017-0229-0

**Published:** 2017-07-31

**Authors:** Ya-Jun Li, Ping-Yong Yi, Ji-Wei Li, Xian-Ling Liu, Tian Tang, Pei-Ying Zhang, Wen-Qi Jiang

**Affiliations:** 1 Department of Lymphoma and Hematology, Hunan Cancer Hospital, 283 Tongzipo Road, Yuelu District, Changsha, 410013 Hunan P. R. China; 2 The Affiliated Cancer Hospital of Xiangya School of Medicine, Central South University, Changsha, 410013 Hunan P. R. China; 3 Cancer Center of the Second Xiangya Hospital, Central South University, Changsha, 410013 Hunan P. R. China; 4 Radioactive Interventional Department, Hunan Cancer Hospital, Changsha, 410013 Hunan P. R. China; 5 Integration Medicine Department, Hunan Chest Hospital, Changsha, Hunan 410013 P. R. China; 6 Department of Medical Oncology, Sun Yat-sen University Cancer Center; State Key Laboratory of Oncology in South China; Collaborative Innovation Center for Cancer Medicine, 651 Dong Feng RD East, Guangdong, 510060 Guangdong P. R. China

**Keywords:** ABO blood type, Extranodal natural killer/T-cell lymphoma, Prognosis, The International Prognostic Index, The Korean Prognostic Index

## Abstract

**Background:**

The prognostic significance of ABO blood type for lymphoma is largely unknown. We evaluated the prognostic role of ABO blood type in patients with extranodal natural killer (NK)/T-cell lymphoma (ENKTL).

**Methods:**

We retrospectively analyzed clinical data of 697 patients with newly diagnosed ENKTL from three cancer centers. The prognostic value of ABO blood type was evaluated using Kaplan–Meier curves and Cox proportional hazard models. The prognostic values of the International Prognostic Index (IPI) and the Korean Prognostic Index (KPI) were also evaluated.

**Results:**

Compared with patients with blood type O, those with blood type non-O tended to display elevated baseline serum C-reactive protein levels (*P* = 0.038), lower rate of complete remission (*P* = 0.005), shorter progression-free survival (PFS, *P* < 0.001), and shorter overall survival (OS, *P* = 0.001). Patients with blood type O/AB had longer PFS (*P* < 0.001) and OS (*P* = 0.001) compared with those with blood type A/B. Multivariate analysis demonstrated that age >60 years (*P* < 0.001), mass ≥5 cm (*P* = 0.001), stage III/IV (*P* < 0.001), elevated serum lactate dehydrogenase (LDH) levels (*P* = 0.001), and blood type non-O were independent adverse predictors of OS (*P* = 0.001). ABO blood type was found to be superior to both the IPI in discriminating patients with different outcomes in the IPI low-risk group and the KPI in distinguishing between the intermediate-to-low- and high-to-intermediate-risk groups.

**Conclusions:**

ABO blood type was an independent predictor of clinical outcome for patients with ENKTL.

## Background

Extranodal natural killer (NK)/T-cell lymphoma (ENKTL), nasal type, is a distinct subtype of non-Hodgkin’s lymphoma (NHL) with unique clinicopathologic characteristics and geographic distribution [1]. ENKTL is relatively prevalent in Asia (e.g., China, Japan, and Korea) and Central/South America (e.g., Mexico and Peru) [2]. ENKTL accounts for 11% of all NHL cases in Chinese populations [3] and accounts for 5%–10% of all NHL cases in the populations of Asia and Central/South America [2, 4]. ENKTL is rarely diagnosed in European and North American populations (e.g., only accounts for 0.2% of NHL cases in the US populations) [5].

The treatment outcomes of ENKTL are generally poor in the CHOP (cyclophosphamide, doxorubicin, vincristine, prednisone) era with 5-year overall survival (OS) rates less than 50% [2, 6–9]. However, the application of l-asparaginase-containing chemotherapy significantly improves the outcomes of ENKTL patients with 5-year OS rate ranging from 50% to 86.9% [6, 10–13]. To date, optimal treatment strategies and prognosis for patients with ENKTL have not been fully defined. Although the prognostic value of the International Prognostic Index (IPI) has been well validated in many subtypes of NHL [14–16], its prognostic value remains controversial in ENKTL [4, 17]. Recently, the prognostic significance of the Korean Prognostic Index (KPI) in ENKTL has been confirmed by several studies, and this model may be further improved by including other laboratory parameters (e.g., C-reactive protein [CRP], albumin levels, and absolute lymphocyte count) [4, 11, 18, 19]. A better understanding of an ideal biomarker that is readily available, inexpensive, and reproducible for ENKTL could improve the prognosis and treatment strategies.

The ABO blood type system was a widely used blood test in clinical practice. Several studies have found that certain ABO blood types are associated with a high risk of certain cancers [20, 21]. Recently, the association between ABO blood type and the survival outcome of cancer patients has also drawn much attention, which has been demonstrated in various solid tumors, including pancreatic cancer [22], colon cancer [23], lung cancer [24], and esophageal cancer [25]. However, to the best of our knowledge, the prognostic value of ABO blood type in lymphoma has never been investigated. We therefore performed this triple-center study to evaluate the prognostic significance of ABO blood type in patients with ENKTL.

## Patients and methods

### Ethics statement

Written informed consent for the patients’ blood samples and other medical information to be stored in our hospitals’ databases and to be used in research were obtained from all patients. This study was approved by the Institutional Review Board of the National Cancer Institute and the ethics committees of Sun Yat-sen University Cancer Center, Hunan Cancer Hospital, and The Second Xiangya Hospital of Central South University. The study was performed in accordance with the Declaration of Helsinki and the institutional guidelines of the local ethics committee.

### Patient selection

We screened consecutive patients with newly diagnosed ENKTL, nasal type, at Sun Yat-sen University Cancer Center, Hunan Cancer Hospital, and The Second Xiangya Hospital of Central South University between January 1998 and June 2015. All of the patients included in this study met the following criteria: (a) pathologically confirmed diagnosis of ENKTL, nasal type, by expert pathologists, according to the World Health Organization (WHO) classification [1], (b) no previous malignancy or any second primary tumor, (c) no previous anti-cancer treatment, (d) available data on ABO blood type, and (e) adequate clinical, laboratory, and follow-up data. Patients with blastic NK-cell lymphoma/leukemia, aggressive NK-cell lymphoma/leukemia, or peripheral T-cell lymphoma, unspecified, were excluded.

All pathologic specimens were reviewed and reclassified by central review, according to the WHO criteria for pathologic diagnosis. Antibodies (Dako, Glostrup, Denmark) to the following antigens were used for immunophenotyping: CD3, CD56, T-cell intracellular antigen (TIA-1), Gram-B, CD45RO, CD20, CD79a, CD30, Ki67, and anaplastic large-cell lymphoma kinase. In situ hybridization was used to detect Epstein–Barr virus (EBV)-encoded RNA.

Before treatment, the following baseline clinical data were collected: patient demographics, physical examinations, Eastern Cooperative Oncology Group performance status (ECOG PS), primary tumor site, B symptoms, treatment modalities and response, ABO blood type, serum lactate dehydrogenase (LDH) level, baseline serum CRP levels, serum EBV-DNA copy number, Ann Arbor stage, and computed tomography (CT) or magnetic resonance (MR) images of the nasopharynx, neck, chest, abdomen, and pelvis or positron emission tomography/computed tomography (PET/CT) images of the entire body. All patients were staged using the Ann Arbor staging system. The IPI (involving age, ECOG PS, stage, LDH level, extranodal sites) and KPI (involving stage, LDH level, B symptoms, regional lymphoma nodes) for nasal NK/T-cell lymphoma were used to perform survival analysis [18, 26].

In addition to the ENKTL patient group, we also selected a hospital-based control group of age- and sex-matched inpatients (case–control ratio = 1) who were diagnosed with nonmalignant disease based on surgical or other clinical management from the above three centers. The number of patients in the control group in each hospital was the same as the corresponding number of patients with ENKTL. If more than one control were matched with a case, one was picked randomly by computer. The data and distribution of ABO antigens in the control group were collected and compared with those of the ENKTL patient group.

### Response criteria and statistical analysis

The response to treatment was assessed according to the International Working Group Recommendations for Response Criteria for non-Hodgkin lymphoma [27]. Progression-free survival (PFS) was defined as the interval between the date of diagnosis and the date of first relapse, progression, death from any cause, or the last date at which the patients were censored. Overall survival (OS) was defined as the duration between the date of diagnosis and either the time of death from any cause or the last date at which patients were censored. The relationships between ABO blood type and clinical and laboratory variables were assessed using Pearson’s Chi square test or Fisher’s test for categorical variables. The log-rank test and Kaplan–Meier method were applied for univariate survival analysis. Variables significant at *P* < 0.05 in univariate analysis were included in multivariate analysis. Multivariate analysis was performed using the Cox proportional hazards model. A two-tailed *P* < 0.05 was considered statistically significant. The statistical software package SPSS 16.0 (SPSS Inc., Chicago, IL, USA) was used for statistical calculations.

## Results

### Patient characteristics

In total, 697 ENKTL patients (492 males and 205 females), with a median age of 43 years (range 10–82 years), met the inclusion criteria. The median ages for blood type O, A, B, and AB groups were 44 years (range 10–80 years), 43 years (range 14–82 years), 42 years (range 16–76 years), and 49 years (range 21–76 years), respectively. The clinical characteristics of the 697 patients are listed in Table 1.

**Table 1:** Basic characteristics of patients with extranodal natural killer (NK)/T-cell lymphoma (ENKTL) in distinct ABO blood type groups

Characteristic	Total (cases)	Blood four-type group [cases (%)]				*P*	Blood two-type group [cases (%)]		*P*
		O	A	B	AB		O	Non-O	
Total	697	255	195	188	59		255	442	
Age (years)						0.313			0.722
≤60	597	220 (86.3)	162 (83.1)	167 (88.8)	48 (81.4)		220 (86.3)	377 (85.3)	
>60	100	35 (13.7)	33 (16.9)	21 (11.2)	11 (18.6)		35 (13.7)	65 (14.7)	
Gender						0.854			0.730
Male	492	178 (69.8)	137 (70.3)	137 (72.9)	40 (67.8)		178 (69.8)	314 (71.0)	
Female	205	77 (30.2)	58 (29.7)	51 (27.1)	19 (32.2)		77 (30.2)	128 (29.0)	
ECOG PS						0.960			0.911
0–1	680	249 (97.6)	190 (97.4)	184 (97.9)	57 (96.6)		249 (97.6)	431 (97.5)	
≥2	17	6 (2.4)	5 (2.6)	4 (2.1)	2 (3.4)		6 (2.4)	11 (2.5)	
B symptoms						0.295			0.133
Yes	324	109 (42.7)	101 (51.8)	86 (45.7)	28 (47.5)		109 (42.7)	215 (48.6)	
No	373	146 (57.3)	94 (48.2)	102 (54.3)	31 (52.5)		146 (57.3)	227 (51.4)	
LDH (U/L)						0.596			0.602
>245	194	68 (26.7)	53 (27.2)	59 (31.4)	14 (23.7)		68 (26.7)	126 (28.5)	
≤245	503	187 (73.3)	142 (72.8)	129 (68.6)	45 (76.3)		187 (73.3)	316 (71.5)	
Tumor size (cm)						0.399			0.953
≥5	65	24 (9.4)	21 (10.8)	18 (9.6)	2 (3.4)		24 (9.4)	41 (9.3)	
<5	632	231 (90.6)	174 (89.2)	170 (90.4)	57 (96.6)		231 (90.6)	401 (90.7)	
Extranodal sites ≥2						0.685			0.966
Yes	77	28 (11.0)	22 (11.3)	18 (9.6)	9 (15.3)		28 (11.0)	49 (11.1)	
No	620	227 (89.0)	173 (88.7)	170 (90.4)	50 (84.7)		227 (89.0)	393 (88.9)	
Regional LN involvement						0.605			0.239
Yes	171	69 (27.1)	42 (21.5)	46 (24.5)	14 (23.7)		69 (27.1)	102 (23.1)	
No	526	186 (72.9)	153 (78.5)	142 (75.5)	45 (76.3)		186 (72.9)	340 (76.9)	
EBV-DNA (copies/mL)^a^						0.435			0.829
<1530	85	31 (50.8)	20 (46.5)	22 (44.9)	12 (66.7)		31 (50.8)	54 (49.1)	
≥1530	86	30 (49.2)	23 (53.5)	27 (55.1)	6 (33.3)		30 (49.2)	56 (50.9)	
CRP (mg/L)^b^						0.197			0.038
≤10	128	55 (61.1)	26 (50.0)	36 (46.3)	11 (42.1)		55 (61.1)	73 (47.1)	
>10	100	35 (38.9)	26 (50.0)	31 (53.7)	8 (57.9)		35 (38.9)	65 (52.9)	
Ann Arbor stage						0.749			0.894
I/II	619	227 (89.0)	173 (88.7)	169 (89.9)	50 (84.7)		227 (89.0)	392 (88.7)	
III/IV	78	28 (11.0)	22 (11.3)	19 (10.1)	9 (15.3)		28 (11.0)	50 (11.3)	
IPI score						0.763			0.421
0–1	597	222 (87.1)	164 (84.1)	162 (86.2)	49 (83.1)		222 (87.1)	375 (84.8)	
2–5	100	33 (12.9)	31 (15.9)	26 (13.8)	10 (16.9)		33 (12.9)	67 (15.2)	
KPI score						0.974			0.984
0–1	478	175 (68.6)	133 (68.2)	128 (68.1)	42 (71.2)		175 (68.6)	303 (68.6)	
2–4	219	80 (31.4)	62 (31.8)	60 (31.9)	17 (28.8)		80 (31.4)	139 (31.4)	

*ECOG PS* Eastern Cooperative Oncology Group performance status, *LDH* lactate dehydrogenase, *LN* lymph node, *EBV* Epstein–Barr virus, *CRP* C-reactive protein, *IPI* International Prognostic Index, *KPI* Korean Prognostic Index

^a^Data of EBV-DNA copy number were available for 171 patients, and the median value was 1530 copies/mL

^b^Data of serum CRP levels were available for 228 patients, and the CRP level >10 mg/L was used as the cutoff value

Most of the patients (680, 97.6%) displayed a favorable performance status (ECOG PS 0–1). Three hundred and twenty-four patients (46.5%) presented with B symptoms. Elevated LDH levels were observed in 194 (27.8%) patients. Sixty-five patients (9.3%) had a mass ≥5 cm, and only 15 (2.2%) displayed bone marrow involvement. One hundred and seventy-one patients (24.5%) displayed regional lymph node involvement, and 77 (11.0%) displayed at least two extranodal involvement sites. Most of the patients (619, 88.8%) had localized disease (stage I/II). According to the IPI, 597 cases (85.7%) were classified as low-risk disease (IPI = 0–1), and 100 (14.3%) were categorized as high-risk disease (IPI = 2–5). The number of patients with KPI = 0–1 was higher than those with KPI = 2–4 [478 (68.6%) vs. 219 (31.4%)]. The baseline CRP levels were available in 228 patients (range 0.16–154.92 mg/L; median value 7.00 mg/L), and the baseline plasma EBV-DNA data were available in 171 patients (range 0–48,500,000 copies/mL; median value 1530 copies/mL).

The distribution of ABO blood types in the ENKTL group was blood type O in 255 (36.6%) patients, blood type A in 195 (28.0%) patients, blood type B in 188 (27.0%) patients, and blood type AB in 59 (8.5%) patients. According to the previous matching method, 697 patients with nonmalignant disease were randomly selected as the control group. The distribution of ABO blood types in the control group was blood type O in 279 (40.0%) patients, blood type A in 175 (25.1%) patients, blood type B in 162 (23.2%) patients, and blood type AB in 81 (11.6%) patients. There was no significant difference in blood type distribution between the ENKTL and control groups (*P* = 0.056). ABO blood type was not associated with patient age, gender, ECOG PS, B symptoms, LDH levels, tumor size, number of extranodal sites, regional lymph node involvement, baseline EBV-DNA copies, Ann Arbor stage, IPI score, or KPI score (all *P* > 0.1, Table 1). However, we found that patients with blood type non-O had a higher percentage of elevated CRP serum level compared with those with blood type O (*P* = 0.038, Table 1).

### Treatment modalities and response

The primary treatment modalities were as follows: 436 (62.6%) patients received chemotherapy combined with radiotherapy, 171 (24.5%) received chemotherapy alone, 72 (10.3%) received radiotherapy alone, and 18 (2.6%) received only best supportive care. The treatment details and responses are listed in Table 2. No significant difference was found in treatment modalities when dividing patient into either four blood type groups (O vs. A vs. B vs. AB, *P* = 0.701) or two groups (O vs. non-O, *P* = 0.690). After the initial treatment, 509 (75.0%) of the 679 treated patients displayed a complete response (CR) or CR unconfirmed (CRu). The rate of CR to initial treatment was significantly higher in the blood type O group than in the blood type non-O group (79.2% vs. 69.5%, *P* = 0.005).

**Table 2:** Primary treatment modalities and responses in patients with ENKTL

Characteristic	Total (cases)	Blood four-type group [cases (%)]				*P*	Blood two-type group [cases (%)]		*P*
		O	A	B	AB		O	Non-O	
Treatment modality						0.701			0.690
CT combined RT	436	164 (64.3)	118 (60.5)	120 (63.8)	34 (57.6)		164 (64.3)	272 (61.5)	
CT alone	171	57 (22.4)	49 (25.1)	47 (25.0)	18 (30.5)		57 (22.4)	114 (25.8)	
RT alone	72	26 (10.2)	23 (11.8)	16 (8.5)	7 (11.9)		26 (10.2)	46 (10.4)	
Best supportive care	18	8 (3.1)	5 (2.6)	5 (2.7)	0 (0)		8 (3.1)	10 (2.3)	
CT regimen						0.675			0.534
CHOP or CHOP-like	218	73 (28.6)	68 (34.9)	61 (32.4)	16 (27.1)		73 (28.6)	145 (32.8)	
EPOCH	105	43 (16.9)	24 (12.3)	32 (17.0)	6 (10.2)		43 (16.9)	62 (14.0)	
ATT	43	15 (5.9)	13 (6.7)	12 (6.4)	3 (5.1)		15 (5.9)	28 (6.3)	
GEMOX + L-asp	232	85 (33.3)	61 (31.3)	60 (31.9)	26 (44.1)		85 (33.3)	147 (33.3)	
SMILE	9	5 (2.0)	1 (0.4)	2 (1.1)	1 (0.4)		5 (2.0)	4 (0.9)	
Complete response	509	202 (79.2)	133 (68.2)	131 (69.7)	43 (72.9)	0.040	202 (79.2)	307 (69.5)	0.005

*CT* chemotherapy, *RT* radiotherapy, *CHOP* cyclophosphamide + doxorubicin + vincristine + prednisone, *EPOCH* etoposide + doxorubicin + vincristine + cyclophosphamide + prednisone, *ATT* alternating triple therapy (*CHOP-B*, cyclophosphamide + doxorubicin + vincristine + bleomycin + prednisone, *IMVP-16* ifosfamide + methotrexate + etoposide; *DHAP*, dexamethasone + cisplatin + cytarabine), *GEMOX* *+* *L-asp* gemcitabine + oxaliplatin + l-asparaginase, *SMILE* dexamethasone + methotrexate + ifosfamide + l-asparaginase + etoposide

### The effect of ABO blood type on survival of patients with ENKTL

**Fig. 1:**
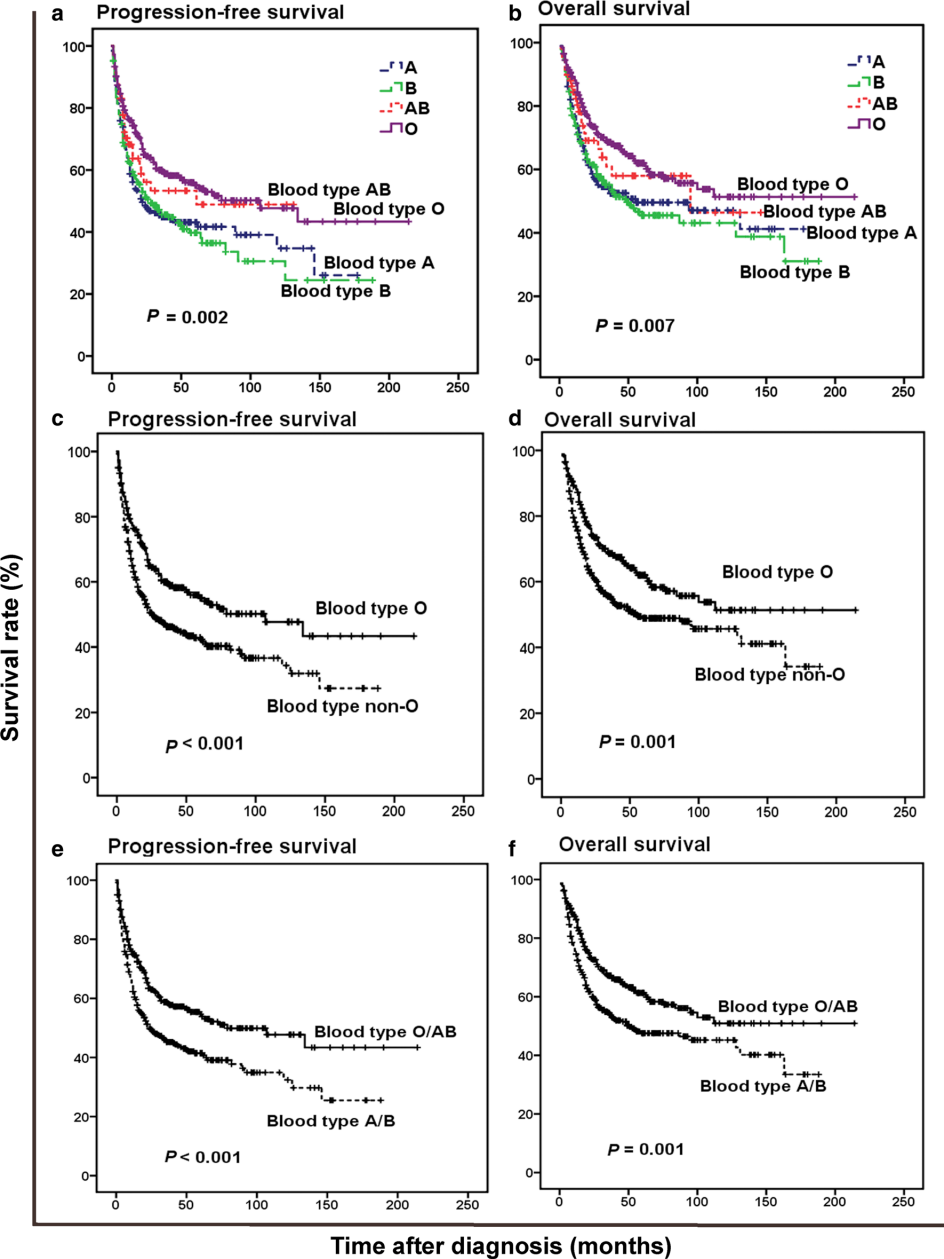
Survival curves of patients with extranodal natural killer (NK)/T-cell lymphoma (ENKTL) according to ABO blood type. **a** Progression-free survival (PFS) curves of patients according to blood types A, B, AB, and O. **b** Overall survival (OS) curves of patients according to blood types A, B, AB, and O. **c** PFS curves of patients according to blood types O and non-O (A, B, and AB). **d** OS curves of patients according to blood types O and non-O. **e** PFS curves of patients according to blood types O/AB versus type A/B. **f** OS curves of patients according to blood types O/AB versus type A/B

There were 302 deaths (43.3%) during a median follow-up of 41 months (range 1–214 months). The deaths were due to tumor progression (*n* = 289), treatment-related toxicities (*n* = 3), cardiovascular diseases (*n* = 2), and unknown causes (*n* = 8). The estimated 5-year PFS and OS rates for all 697 patients were 47.3% and 53.6%. The 5-year PFS rates for blood type A, B, AB, and O groups were 43.1%, 39.7%, 53.3%, and 55.0%, respectively (*P* = 0.002, Fig. 1a). The 5-year OS rates were 49.6%, 45.5%, 58.0%, and 62.0%, respectively (*P* = 0.007, Fig. 1b). Because the ENKTL patients with blood type O had longer PFS and OS compared with those with blood type A, B, or AB, we therefore divided these patients into blood type O and non-O (A, B, and AB) groups. The patients with blood type O had significantly higher 5-year PFS rate (55.0% vs. 42.9%, *P* < 0.001, Fig. 1c) and 5-year OS rate (62.0% vs. 48.9%, *P* = 0.001, Fig. 1d) compared with those with blood type non-O. Since patients with blood type AB and O have similar PFS and OS, we next examined the effect of ABO blood type on survival by comparing type O/AB versus type A/B. We found that patients with blood type O/AB had significantly higher 5-year PFS rate (54.6% vs. 41.5%, *P* < 0.001, Fig. 1e) and 5-year OS rate (61.3% vs. 47.6%, *P* = 0.001, Fig. 1f) compared with those with blood type A/B.

Blood type non-O was significantly associated with shorter OS in patients with Ann Arbor stage I/II disease (*P* = 0.002), but not in advanced cases (*P* = 0.151). For patients receiving chemotherapy plus radiotherapy, blood type non-O was also significantly associated with shorter OS (*P* = 0.003), whereas among patients receiving chemotherapy or radiotherapy alone, blood type non-O did not significantly affect the survival (*P* = 0.158 and *P* = 0.352, respectively). Blood type non-O was also associated with a worse outcome among patients receiving anthracyclines-containing chemotherapy (*P* = 0.010) or l-asparaginase-containing chemotherapy (*P* = 0.011). Table 3 displays the detailed data of prognostic significance of ABO blood type (O vs. non-O) in different subgroups.

**Table 3:** The prognostic significance of ABO blood type in different subgroups of ENKTL patients

Subgroup	5-year OS rate (%)		*P*
	Blood type O	Blood type non-O	
Ann Arbor stage			
I/II	66.1	53.2	0.002
III/IV	30.6	12.1	0.151
Treatment modality			
CT combined RT	74.5	57.2	0.003
CT alone	37.7	30.2	0.158
RT alone	56.7	55.5	0.352
Chemotherapy regimen			
Anthracyclines-containing	59.4	42.8	0.010
l-Asparaginase-containing	80.6	62.3	0.011

*OS* overall survival, *CT* chemotherapy, *RT* radiotherapy

### Univariate and multivariate Cox regression analysis

Table 4 displays the results of the univariate and multivariate analysis of potential predictors of PFS and OS. Multivariate analysis using the forward conditional Cox region model identified tumor size ≥5 cm (risk ratio [RR] = 1.555, 95% confidence interval [CI] 1.130–2.139, *P* = 0.007), regional lymph node involvement (RR = 1.323, 95% CI 1.041–1.680, *P* = 0.022), blood type non-O (RR = 1.539, 95% CI 1.266–1.932, *P* < 0.001), and an IPI score ≥2 (RR = 2.285, 95% CI 1.751–2.983, *P* < 0.001) as adverse factors for PFS. In the multivariate analysis for OS, age >60 years (RR = 1.904, 95% CI 1.434–2.527, *P* < 0.001), tumor size ≥5 cm (RR = 1.720, 95% CI 1.233–2.400, *P* = 0.001), stage III/IV (RR = 2.114, 95% CI 1.554–2.875, *P* < 0.001), elevated LDH levels (RR = 1.514, 95% CI 1.183–1.936, *P* = 0.001), and blood type non-O (RR = 1.491, 95% CI 1.166–1.906, *P* = 0.001) were significant independent predictors of OS.

**Table 4:** Univariate and multivariate analysis of prognostic factors for PFS and OS in patients with ENKTL

Variable	PFS			OS		
	Univariate analysis	Multivariate analysis		Univariate analysis	Multivariate analysis	
	*P*	RR (95% CI)	*P*	*P*	RR (95% CI)	*P*
Age (>60 years vs. ≤60 years)	0.002	1.140 (0.886–1.467)	0.308	<0.001	1.904 (1.434–2.527)	<0.001
B symptoms (yes vs. no)	0.052	–	–	0.019	1.285 (0.981–1.685)	0.069
Tumor size ≥5 cm (yes vs. no)	<0.001	1.555 (1.130–2.139)	0.007	<0.001	1.720 (1.233–2.400)	0.001
Extranodal sites ≥2 (yes vs. no)	<0.001	0.974 (0.577–1.642)	0.920	<0.001	0.857 (0.491–1.496)	0.586
Regional LN involvement (yes vs. no)	<0.001	1.323 (1.041–1.680)	0.022	0.030	1.230 (0.898–1.686)	0.197
Stage III/IV (yes vs. no)	<0.001	1.142 (0.650–2.009)	0.644	<0.001	2.114 (1.554–2.875)	<0.001
LDH >245 U/L (yes vs. no)	<0.001	1.293 (0.937–1.783)	0.118	<0.001	1.514 (1.183–1.936)	0.001
Blood group non-O (yes vs. no)	<0.001	1.539 (1.266–1.932)	<0.001	0.001	1.491 (1.166–1.906)	0.001
IPI score ≥2 (yes vs. no)	<0.001	2.285 (1.751–2.983)	<0.001	<0.001	1.021 (0.570–1.828)	0.945
KPI score ≥2 (yes vs. no)	<0.001	0.970 (0.642–1.466)	0.886	<0.001	0.846 (0.539–1.327)	0.467

*PFS* progression-free survival, *OS* overall survival, *RR* relative risk, *CI* confidence interval, *LN* lymph node, *LDH* lactate dehydrogenase, *IPI* International Prognostic Index, *KPI* Korean Prognostic Index, *–* not assessed

### The comparison of prognostic value between ABO blood type and IPI and KPI models

**Fig. 2:**
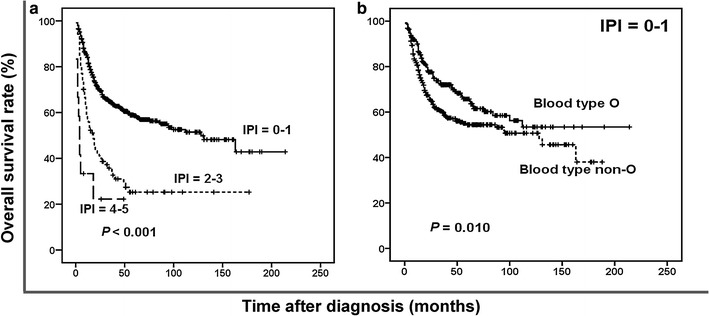
Survival curves of patients with ENKTL according to the International Prognostic Index (IPI) score. **a** OS curves of patients according to the IPI. **b** OS curves of patients with IPI score = 0–1 according to blood types O and non-O

Using the IPI predictive model, we identified 3 categories of patients with different survival outcomes: 598 (85.8%) patients in the low-risk (IPI = 0–1) group, 87 (12.5%) in the intermediate-risk (IPI = 2–3) group, and 12 (1.7%) in the high-risk (IPI = 4–5) group. The 5-year OS rate was 58.6% for the low-risk group, 25.3% for the intermediate-risk group, and 22.2% for the high-risk group (*P* < 0.001, Fig. 2a). Significant differences in survival were also found between the low-risk and intermediate-risk groups (*P* < 0.001) and between the intermediate-risk and high-risk groups (*P* = 0.024). However, based on the IPI data, 85.8% of the patients were disproportionately grouped into the low-risk group, and the IPI score was unable to identify patients with different survival statuses within the low-risk group, whereas ABO blood type (O vs. non-O) efficiently categorized patients in the low-risk IPI group into two subgroups with different survival outcomes (*P* = 0.010, Fig. 2b).

**Fig. 3:**
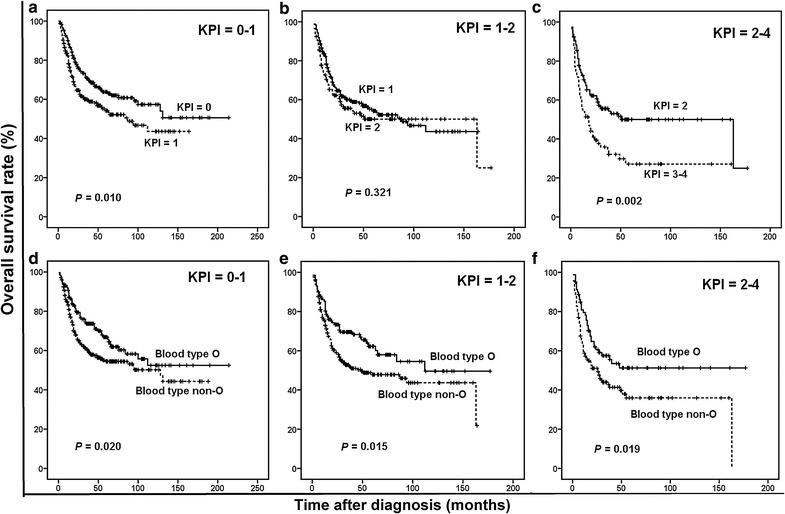
Survival curves of patients with ENKTL according to the Korean Prognostic Index (KPI) score. **a** OS curves of patients with KPI score = 0–1 according to the KPI model. **b** OS curves of patients with KPI score = 1–2 according to the KPI model. **c** OS curves of patients with KPI score = 2–4 according to the KPI model. **d** OS curves of patients with KPI score = 0–1 according to blood types O and non-O. **e** OS curves of patients with KPI score = 1–2 according to blood types O and non-O. **f** OS curves of patients with KPI score = 2–4 according to blood types O and non-O

The KPI model balanced the distribution of patients in different risk categories more efficiently than the IPI model (score 0:233 cases, 33.4%; score 1:245 cases, 35.2%; score 2:145 cases, 20.8%; and score 3–4:74 cases, 10.6%), and it was able to discriminate between patients with different survival outcomes. The 5-year OS rate was 63.8% for the KPI = 0 group, 54.2% for the KPI = 1 group, 50.0% for the KPI = 2 group, and 27.1% for the KPI = 3–4 group (*P* < 0.001). Moreover, the KPI model significantly distinguished between the low- and intermediate-to-low-risk groups (KPI = 0 vs. KPI = 1, *P* = 0.010, Fig. 3a), but not between the intermediate-to-low- and high-to-intermediate-risk groups (KPI = 1 vs. KPI = 2, *P* = 0.321, Fig. 3b). The KPI model also significantly distinguished between the high-to-intermediate- and high-risk groups (KPI = 2 vs. KPI = 3–4, *P* = 0.002, Fig. 3c). In contrast, ABO blood type (O vs. non-O) was efficient at discriminating patients with a KPI score = 0–1 (*P* = 0.020, Fig. 3d), 1–2 (*P* = 0.015, Fig. 3e), or 2–4 (*P* = 0.019, Fig. 3f).

## Discussion

In the present triple-center study, we found that ABO blood type (O vs. non-O) is an independent prognostic factor of PFS and OS and a useful predictor of treatment response in patients with ENKTL. Moreover, the proportions of blood types O, A, B, and AB were similar to those reported previously for Chinese patients with solid tumors [23, 24]. However, because no studies published in English to date have investigated the prognostic role of ABO blood type on the outcome of lymphoma, we could not compare our results with published information. Despite this, the results of the current study are consistent with several previous studies in which blood type O was reported as a favorable prognostic factor for various types of solid tumors [22–25, 28, 29]. Rahbari et al. [29] investigated the influence of ABO blood type on 627 patients with pancreatic cancer and found blood type O as a favorable prognostic factor (hazard ratio [HR] = 0.78, *P* = 0.037). Similarly, Li et al. [24] evaluated the prognostic role of ABO blood type in 1601 patients with curatively resected non-small cell lung cancer (NSCLC), and they found that patients with blood type O or B had significantly prolonged OS and disease-free survival (DFS) compared with those with blood type A or AB. Moreover, in the study of Gershman et al. [28] of a large cohort of 2086 patients with bladder cancer, the results indicated that blood type non-O was associated with significantly shorter recurrence-free survival (RFS, *P* = 0.04) and cancer-specific survival (CSS, *P* = 0.02). In contrast, some studies did not find any effect of ABO blood type on survival outcome in some other cancers [30, 31]. Gates et al. [30] evaluated the impact of ABO blood type on the survival of 2036 invasive breast cancer patients and found no association. In addition, Lee et al. [31] found that ABO blood type was not associated with survival outcomes and was not a prognostic factor in patients who underwent surgery for renal carcinoma. Thus, further studies with larger sample sizes are warranted to confirm the prognostic role of ABO blood type in patients with ENKTL.

Moreover, in the present study, we found that the significant differences in PFS and OS holds true not only when patients were grouped into blood types O and non-O but also when patients were grouped into blood types O/AB and A/B. In addition, we observed longer survival in patients with blood type AB than in those with blood type A/B, although the difference was not significant (data not shown). It seems that blood type AB is a favorable factor for ENKTL. Our results are consistent with a previous study in which blood type AB was reported as a favorable prognostic factor for colon cancer [23]. In contrast, some studies found that blood type AB was an unfavorable prognostic factor for several other types of cancer [24, 25]. Because the number of patients with blood type AB is the lowest among all blood types, the influence of blood type AB on the prognostic value of ABO blood type in ENKTL may be rather small. More studies are needed to evaluate the prognostic role of blood type AB.

In our study, the prognostic significance of ABO blood type was observed not only in the entire cohort analysis but also in subgroup analysis. Blood type non-O was significantly associated with shorter OS as compared with blood type O in patients with Ann Arbor stage I/II disease, patients receiving chemotherapy plus radiotherapy, patients receiving anthracyclines-containing chemotherapy, or patients receiving l-asparaginase-containing chemotherapy. The prognostic value of the IPI score has been widely validated for diffuse large B-cell lymphoma (DLBCL) and many other subtypes of NHL. However, its prognostic role in ENKTL remains controversial, and the patients were allocated disproportionately by the IPI model [4, 17]. In the present study, although the IPI score was significantly predictive in the univariate analysis, it failed to identify patients with varying survival rates within the low-risk group, which accounted for a majority of the patients. When ABO blood type was added to the IPI model, the low-risk patients were separated into two groups with significantly different survival outcomes. The KPI model yielded a balanced distribution of patients with different levels of risk and separated them into four groups with different survival outcomes. However, the KPI model failed to significantly distinguish between the intermediate-to-low- and high-to-intermediate-risk groups. Conversely, ABO blood type was able to discriminate between the intermediate-to-low- and high-to-intermediate-risk groups, between the low- and intermediate-to-low-risk groups, and between the high-to-intermediate- and high-risk groups. Taken together, these results indicated that ABO blood type has a powerful prognostic value. Further studies are required to determine whether ABO blood type can improve the currently widely used KPI model in patients with ENKTL.

The mechanisms underlying the influence of ABO blood type on cancer development and progression are still largely unclear. However, several potential explanations have been proposed for the association of ABO blood type and cancer. Modified expression of blood group antigens on cancer cells has been hypothesized to influence the development and spread of cancer, by altering glycosyltransferase specificity or by altering cell motility, resistance to apoptosis, and immune escape [32]. Moreover, variations in the surface antigen expression on the surrounding epithelial and endothelial cells would affect the adhesion and signaling of cancer cells [33]. Other recent studies demonstrated that ABO blood type is associated with serum levels of soluble intercellular adhesion molecule-1 (sICAM-1), tumor necrosis factor-alpha (TNF-α), P-selectin, and soluble E-selectin, suggesting that blood group antigens may influence the chronic systemic inflammatory response, which is associated with the processes of angiogenesis, tumor growth, invasion, and migration [34, 35]. In addition, the present study found that the patients with blood type non-O had a higher percentage of elevated CRP serum level compared with those with blood type O. CRP is an acute-phase protein secreted by hepatocytes during the inflammatory response, and it is regulated by pro-inflammatory cytokines [36]. This finding suggests that ABO blood type may affect the prognosis of patients with ENKTL by changing the concentration of inflammatory cytokines. Moreover, multiple previous studies demonstrated that blood type non-O was independently associated with a risk of venous thromboembolism (VTE) [37, 38]. Although there was no data on the association between ABO blood type and VTE in patients with lymphoma, Mizrahi et al. [38] found that blood type non-O was an independent risk factor for VTE in children with acute lymphoblastic leukemia. In the current study, we indeed found that two patients, whose ABO blood types were A and B, died from acute pulmonary thromboembolism. Based on these data, we speculate that an increasing death rate caused by thromboembolism is another possible mechanism underlying the relationship between blood type non-O and poor prognosis.

We acknowledge some important limitations of our study. First and foremost, the study design was retrospective and non-randomized. Second, the relatively small sample size prevented us from statistically analyzing the survivor factors. Third, the therapeutic heterogeneity of the treatment strategy of patients may confound the results of our study.

## Conclusions

In conclusion, our study showed an association between ABO blood type and survival in patients with lymphoma. We found that ABO blood type is an independent predictor of clinical outcome for ENKTL patients. Further studies with a large series and more uniform treatment are warranted to confirm the prognostic value of ABO blood type in patients with ENKTL.

## Abbreviations

ENKTL: extranodal natural killer (NK)/T-cell lymphoma
NHL: non-Hodgkin’s lymphoma
PFS: progression-free survival
OS: overall survival
IPI: International Prognostic Index
KPI: Korean Prognostic Index
LDH: lactate dehydrogenase
CRP: C-reactive protein
RT: radiotherapy
CR: complete response
RR: relative risk
CI: confidence interval
TNF: tumor necrosis factor-alpha
VTE: venous thromboembolism
ALL: acute lymphoblastic leukemia
PTE: pulmonary thromboembolism
